# Demonstration of MOCVD-grown Ga_2_O_3_ power MOSFETs on sapphire with in-situ Si-doped by tetraethyl orthosilicate (TEOS)

**DOI:** 10.1186/s11671-023-03858-w

**Published:** 2023-05-30

**Authors:** Sao Thien Ngo, Chan-Hung Lu, Fu-Gow Tarntair, Sheng-Ti Chung, Tian-Li Wu, Ray-Hua Horng

**Affiliations:** 1 International College of Semiconductor Technology, National Yang Ming Chiao Tung University, Hsinchu, 30010 Taiwan; 2 Institute of Electronics, National Yang Ming Chiao Tung University, Hsinchu, 30010 Taiwan

**Keywords:** Ga_2_O_3_ power MOSFETs, In-situ Si doping, MOCVD, Sapphire substrate

## Abstract

In this work, we demonstrated Ga_2_O_3_-based power MOSFETs grown on c-plane sapphire substrates using in-situ TEOS doping for the first time. The *β*-Ga_2_O_3_:Si epitaxial layers were formed by the metalorganic chemical vapor deposition (MOCVD) with a TEOS as a dopant source. The depletion-mode Ga_2_O_3_ power MOSFETs are fabricated and characterized, showing the increase of the current, transconductance, and breakdown voltage at 150 °C. In addition, the sample with the TEOS flow rate of 20 sccm exhibited a breakdown voltage of more than 400 V at RT and 150 °C, indicating that the in-situ Si doping by TEOS in MOCVD is a promising method for Ga_2_O_3_ power MOSFETs.

## Introduction

β-Ga_2_O_3_ is an emerging material as a potential candidate of high-power device application due to its ultra-wide bandgap of about 4.8 eV [1]. Furthermore, the critical electric field (E_c_) of the β-Ga_2_O_3_ up to 8 MV/cm is much higher than those common high-power materials such as 4H-SiC (2.5 MV/cm) and GaN (3.3 MV/cm) [2]. It results in the Baliga figure of merit (BFOM) of the β-Ga_2_O_3_ being 3214 which is ten times of 4H-SiC (317) and four times of GaN (846) [3]. In addition, the critical problem for SiC and GaN-based devices is a high thermal budget for their crystal growth while the growth temperature of β-Ga_2_O_3_ is much lower than those materials and Ga_2_O_3_ could be grown epitaxy film using different of methods [4]. The method of metalorganic chemical vapor deposition (MOCVD) can be used to mass product and to grow the Ga_2_O_3_ on sapphire substrate [5]. The MOCVD-grown epitaxial layer of Ga_2_O_3_ could be doped during growth process by tetraethyl orthosilicate (TEOS) which was first demonstrated in one of the studies [6]. Ga_2_O_3_ is also known for the availability of high-quality, large-diameter single-crystal substrate which is grown directly from the melt, with much lower growth temperature and lower cost in process [7, 8].

Those amazing material characteristics mentioned above have been reported for many electrical devices like metal–oxide–semiconductor field-effect transistors (MOSFET) [9–11], metal–semiconductor field-effect transistors (MESFET) [12], and Schottky barrier diode [13]. However, most of the devices are grown homoepitaxially and doped by the Si-ion implantation as a shallow donor to increase the conductivity [1].

Here, we reported, for the first time, the depletion (D)-mode MOSFETs by grown heteroepitaxy β-Ga_2_O_3_ on sapphire with in-situ Si doped by MOCVD. The performance of D-mode MOSFETs with different in-situ Si-doped concentration will be studied. Moreover, the device performance operated at high temperature will also be discussed.

## Device structure and fabrication

The n-type β-Ga_2_O_3_ epilayers with 200-nm thickness were grown on c-plane sapphire substrate at 875 °C by MOCVD. The growth pressure was 25 Torr. Triethylgallium (TEGa) and O_2_ with the flow rate of 100 sccm and 500 sccm were used as the precursors for Ga and O [14]. The current growth process has been optimized. Detailed crystallinity of the Ga_2_O_3_ has been studied in our previous publication [15]. The tetraethyl orthosilicate (TEOS) precursor used the n-type dopant source. To study the effect of different doped active layers on the performance of depletion (D)-mode MOSFET, the β-Ga_2_O_3_ epilayer was doped by TEOS with 10 and 20 sccm and denoted A and B samples, respectively. Before the device fabrication, the material characteristics of sample A and B have measured by secondary ion mass spectrum (SIMS) and Hall measurements.

**Fig. 1:**
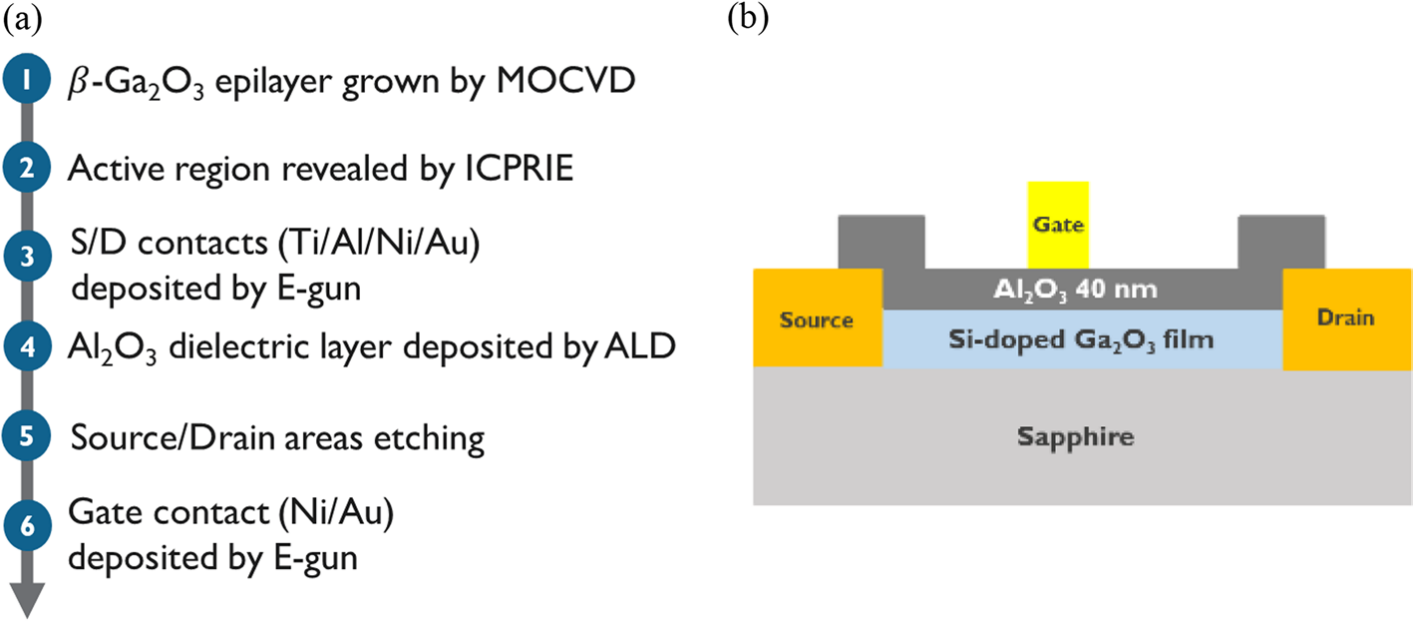
**a** Fabrication process flow and **b** schematic cross-sectional structure of the depletion-mode Ga_2_O_3_-based MOSFET devices

Then, the active region was defined by the etching mesa by inductively coupled plasma reactive ion etching (ICPRIE) using Ar and Cl_2_. After, Ti/Al/Ni/Au (20/100/40/50 nm) was deposited by E-gun deposition system as the Source (S) and drain (D) Ohmic contact electrodes. The specific contact resistance (${\uprho }_{{\text{C}}}$) was measured to be 10^8^ Ω-cm^2^ on the transfer length structure. After ohmic metal deposition, the 40 nm Al_2_O_3_ dielectric layer was deposited by atomic layer deposition (ALD) as the gate oxide. Regarding the pre-treatment before Al_2_O_3_ deposition, the acetone, IPA, and DI water were used to clean wafers. Noted that the traditional RCA clean for our device was not used because the HF contained in the RCA clean process may etch the Ga_2_O_3_ epilayer. The Picosun R-200 was used to grow the Al_2_O_3_ dielectric layer at 250 °C by thermal deposition. The H_2_O and TMA were used as the precursors. The first pulse time was 0.1 s to let the H_2_O have self-limiting reaction with Ga_2_O_3_ film and terminated when the surface ran out of reactant. The excess gas would be purged in 8 s by N_2_. Then the second reactant TMA will be introduced into chamber with same pulse time and purge time, again reacting with surface species, concluding one cycle. We processed 353 cycles to deposit our dielectric layer. Then, the ohmic contact region (Source/Drain) was opened by ICPRIE etching. Finally, the Ni/Au (30/150 nm) gate (G) metal was deposited by E-gun deposition system. The MOSFETs have a gate length 4 um (L_G_), a gate-to-source width (L_GS_) 7 um, and a gate-to-drain width (L_GD_) 9 um. Figure 1 shows the schematic structure of the D-mode Ga_2_O_3_-based MOSFETs fabricated on sapphire substrate. The key processes of TEOS parameters are summarized in Table 1.

**Table 1:** Heteroepitaxial growth conditions

Sample	Ga/O/TEOS flow rate (sccm)	Si con. measured by SIMS (1/cm^3^)	Carrier con. measured by Hall (1/cm^3^)	Mobility (cm^2^/V s)
A	100/1000/10	5.5 × 10^19^	6.49 × 10^17^	0.689
B	100/1000/20	1.1 × 10^20^	3.2 × 10^18^	0.144

## Results and discussion

**Fig. 2:**
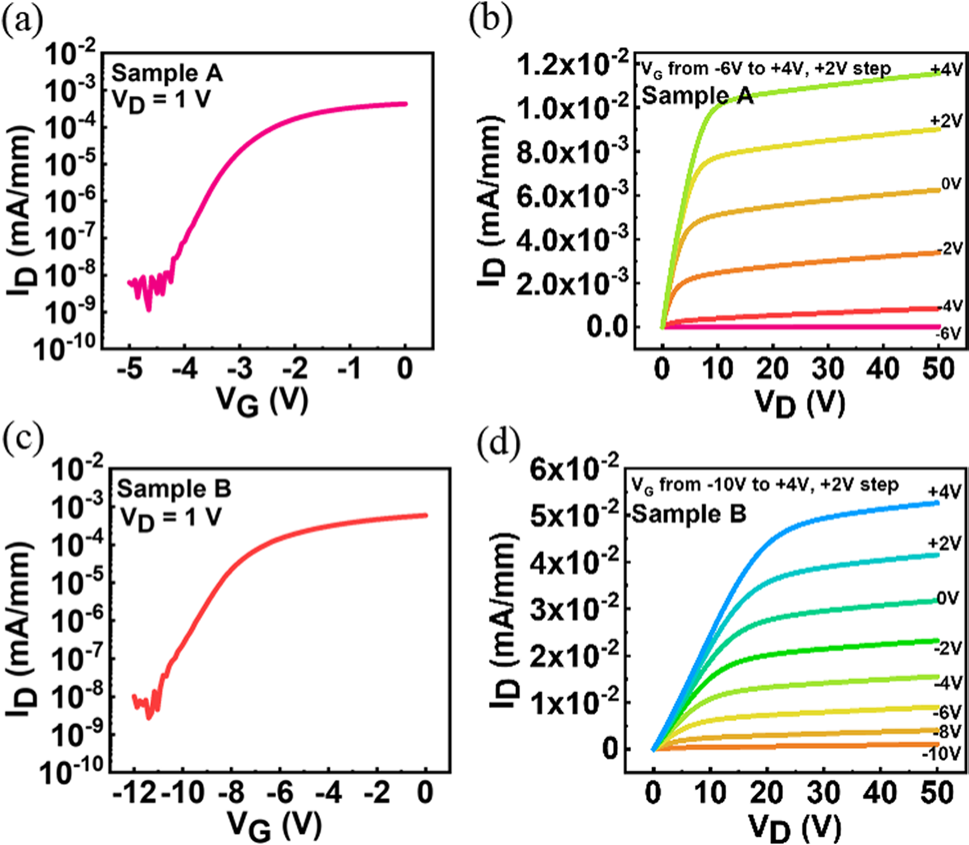
Transfer characteristics of **a** sample A **b** sample B and output characteristics of **c** sample A **d** sample B

In this MOSFET structure, only one Si-doped Ga_2_O_3_ epilayer was used as an active layer. The Si concentration, carrier concentration and mobility shown in Table 1 for samples A and B were measured by SIMS and Hall effect system. The Si concentrations were 5.5 × 10^19^ and 1.1 × 10^20^/cm^3^ for the TEOS flow rate of 10 and 20 sccm, respectively. The corresponding carrier concentrations were 6.49 × 10^17^ and 3.2 × 10^18^/cm^3^ and mobility were 0.689 and 0.144 cm^2^/V s for the TEOS flow rate of 10 and 20 sccm. The low mobility could be resulted from the high Si concentration and thin epilayer. Even though, it is important to evaluate the performance of the device. Figure 2. shows the I_D_–V_G_ and I_D_–V_D_ characteristics in A and B samples, demonstrating the well-behaved performances with the increase of drain current as the TEOS flow rate increases. Meanwhile, the threshold voltage shifts to more negative in the case of sample B. Table 2 summarizes the key electrical characteristics in sample A and B recorded at V_D_ = 20 V and V_G_ = 4 V.

**Table 2:** Sample A, B with drain saturation current and threshold voltage values

TEOS flow rate (sccm)	I_D,sat_ (mA/mm)	V_TH_ (V)
10	0.011	− 3.4
20	0.044	− 8.1

**Fig. 3:**
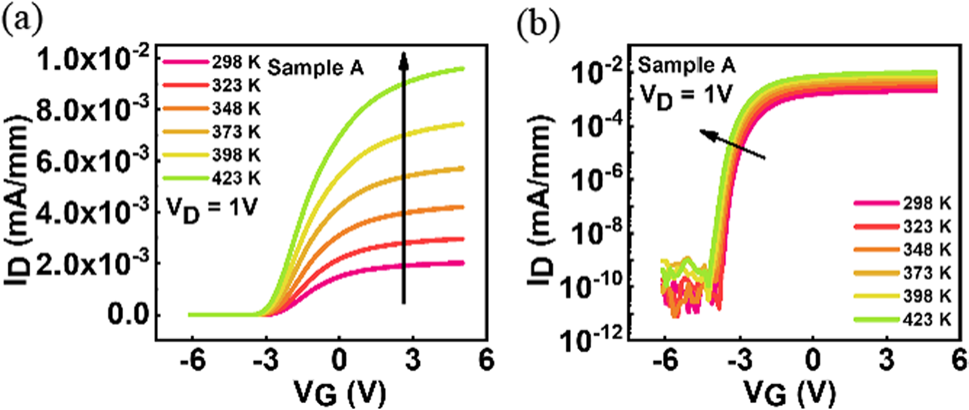
Temperature-dependent I_D_–V_G_ characteristics of sample A with TEOS flow rate of 10 sccm recorded at V_D_ = 1 V

**Fig. 4:**
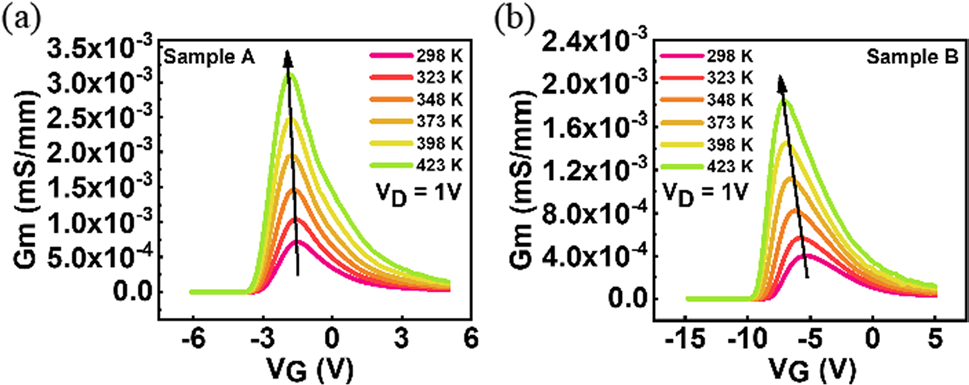
Temperature-dependent I_D_-V_G_ characteristics of sample B with TEOS flow rate of 20 sccm recorded at V_D_ = 1 V

The high temperature characteristics of Ga_2_O_3_-based MOSFETs are also investigated. Figures 3 and 4. show the I_D_–V_G_ characteristics with different temperature, showing an increase of the drain current as the temperature increases, which is consistent with the observation in some recent studies [16, 17].

**Fig. 5:**
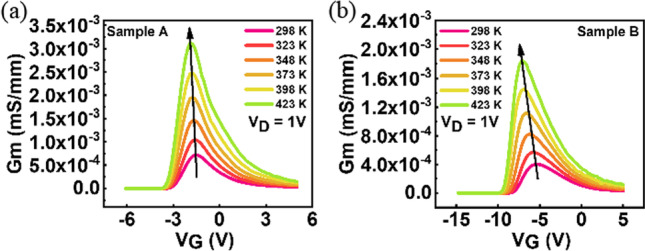
Transconductance extraction from the I_D_-V_G_ curves recorded at V_D_ = 1 V of **a** sample A and **b** sample B

The transconductances of both samples are extracted and shown in Fig. 5. The transconductance increases and shifts to more negative gate bias as temperature increases. The samples with lower TEOS flow rate show higher g_m_ peaks, suggesting a high electron mobility in sample A.

**Fig. 6:**
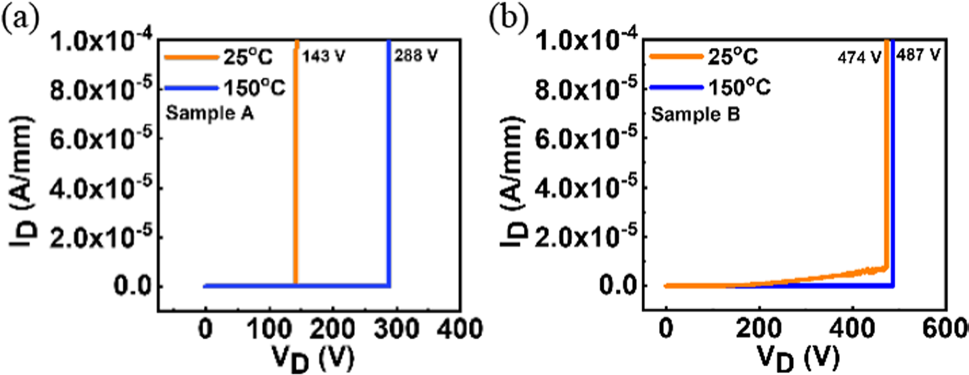
The breakdown characteristics of **a** sample A and **b** sample B under RT and 150 °C

The breakdown characteristics (Fig. 6) are performed with a Keysight B1505 device analyzer. Sample A shows the breakdown voltage of 143 V at 25 °C and 288 V at 150 °C and sample B shows the breakdown voltage of 474 V at 25 °C and 487 V at 150 °C, respectively, indicating that high TEOS flow during MOCVD process can enhance the breakdown voltage. Besides, a positive temperature dependency of the breakdown voltage is both observed in sample A and sample B, which is most probably due to the impact ionization [18]. Table 3 shows the benchmark of the breakdown voltage in this work compared to others, clearly indicating that growing Ga_2_O_3_ using MOCVD with the doping by TEOS is promising for power electronics application.

**Table 3:** Benchmark of some key parameters in Ga_2_O_3_ FETs on native and sapphire substrate

Material growth	Substrate	L_GD_ (um)	n_e_ (cm^−3^)	V_br_ (V)		Reference
MOCVD (in-situ Si doping by TEOS)	Sapphire	9	6.5 × 10^17^	143 (298 K)	288 (423 K)	This work
			3.2 × 10^28^	474 (298 K)	487 (423 K)	This work
MOCVD (doping by Silane)	Sapphire	20	2 × 10^18^	390 (300 K)		[16]
MOCVD (doping by Silane)	Sapphire	20	1.05 × 10^16^	400 (300 K)		[5]
MOCVD	$\beta$-Ga_2_O_3_	6	3 × 10^17^	1200 (300 K)		[14]
MBE	$\beta$-Ga_2_O_3_	8	3 × 10^17^	400 (300 K)	370 (523 K)	[9]
MBE	$\beta$-Ga_2_O_3_	17.5	3.3 × 10^13^	51 (300 K)		[19]
HVPE	$\beta$-Ga_2_O_3_		1.2 × 10^16^	961 (300 K)		[20]

n_e_: Carrier concentration in Ga_2_O_3_:Si thin films

## Conclusion

In conclusion, the Ga_2_O_3_ power MOSFETs with the MOCVD-grown epitaxial layer and in-situ Si doping on sapphire substrate are demonstrated for the first time. Firstly, the depletion-mode Ga_2_O_3_ power MOSFETs are fabricated and characterized in the different temperature, showing the high saturation current and high transconductance as temperature increases. Furthermore, the devices with TEOS of 20 sccm (sample B) can achieve more than 400 V breakdown voltage at RT and 150 °C. Lastly, the Ga_2_O_3_ power MOSFETs with in-situ Si doping using TEOS show promising breakdown voltage characteristics compared to the recent works. In sum, we demonstrate that TEOS doping is promising for high-performance Ga_2_O_3_ power MOSFETs.

## Data Availability

The datasets generated during and/or analysed during the current study are available from the corresponding author on reasonable request.
